# Acquired methemoglobinemia from phenazopyridine use

**DOI:** 10.1186/s12245-018-0208-5

**Published:** 2018-11-12

**Authors:** Travis Murphy, Melinda Fernandez

**Affiliations:** University of Florida Emergency Medicine Residency, Suite 5270, 1329 SW 16th St, Gainesville, FL 32610 USA

**Keywords:** Acquired methemoglobinemia, Toxicology, Community emergency medicine

## Abstract

**Background:**

Phenazopyridine-induced methemoglobinemia is relatively rare with fewer than ten cases reported over the past 35 years. We describe a case of phenazopyridine-induced methemoglobinemia that is unique in the way the patient presented and how initial workup was completed.

**Case presentation:**

The patient presented with lethargy, diarrhea, light-headedness, and headaches, with past medical history of breast cancer, seizures, and recent dysuria for which she had been taking phenazopyridine. She was noted to have a persistent hypoxemia despite supplemental oxygen delivery and a shunting process was considered, with pulmonary embolus and methemoglobinemia due to phenazopyridine use being of chief concern. She was stabilized, and confirmation of original diagnosis was made at the main ED and treatment successfully rendered with good effect.

**Conclusions:**

Methemoglobinemia, while rare, can be associated with use of over-the-counter medicines and should be considered as part of a broad differential. This case serves to emphasize the importance of a thorough history and physical—tools especially helpful when at a satellite facility with relatively limited resources.

## Background

Phenazopyridine is a commonly used over-the-counter medication for treatment of symptoms associated with urinary tract infections. In medical education, phenazopyridine is considered a classic cause of drug-induced methemoglobinemia. However, phenazopyridine-induced methemoglobinemia is relatively rare with fewer than ten cases reported over the past 35 years [1–5]. We describe a case of phenazopyridine-induced methemoglobinemia that is unique for three reasons. First, the initial clinical presentation was suggestive of other, common causes of hypoxia. Second, the clinical presentation differs from the few cases of phenazopyridine-induced methemoglobinemia reported in the medical literature. Finally, in this case, the patient presented to a free-standing emergency department (ED) with limited diagnostic and treatment capability compared to a hospital-based facility. However, with thorough history-taking and physical exam, the correct diagnosis was suspected and decision to treat was made after ruling out pulmonary embolism upon arrival to the hospital.

## Case presentation

A 57-year-old female with medical history significant for seizures and breast cancer status-post mastectomy presented to a free-standing ED with chief complaint of headaches, lethargy, diarrhea, and light headedness. She had been treated with neoadjuvant therapy for the breast cancer 12 years prior to admission and was in remission at the time of presentation. The patient reported dysuria, urinary frequency, and urgency 2 days prior to admission for which she took over-the-counter phenazopyridine for symptom relief. Over the subsequent 48 h, she took approximately 24 tablets for persistent symptoms, and started to develop a headache, lethargy, and light-headedness, prompting the visit to the free-standing ED. The patient denied fevers, productive sputum, or recent changes in her regular medications apart from starting the phenazopyridine. Her medications included amitriptyline, cetirizine, clonazepam, diclofenac gel, fluticasone, pramipexole, pregabalin, and vitamin D supplements.

While in the ED, the patient was noted to be tachycardic to 100–110, blood pressure 146/77, and respiratory rate 18 with clear breath sounds bilaterally, but pale and cyanotic appearing lips, hands, toes, with pulse oximeter readings of 80–88%. Chest X-ray did not show focal infiltrates. A non-contrasted head CT scan was negative. A basic metabolic panel was unremarkable, the white blood cell count was normal, but the hemoglobin was 10.5 g/dl and hematocrit 33.6%. The patient was placed on supplemental oxygen via nasal cannula with no change in her O_2_ saturations via pulse oximetry. Urinalysis showed positive white blood cells and was noted to be a deep red-orange color (attributed to the phenazopyridine).

Given continued hypoxia despite supplemental oxygen administration and in the context of phenazopyridine use, prior malignancy, and lack of current anticoagulation, drug-induced methemoglobinemia and pulmonary embolus were considered the most likely diagnoses. An arterial blood gas (ABG) was obtained and revealed a PO_2_ 151 mmHg and arterial O_2_ saturation of 99% making methemoglobinemia the most likely diagnosis. The blood was noted to be dark. This free-standing ED does not have capabilities to test co-oximetry and did not have methylene blue available, so the patient was transferred to the hospital-based ED where she was found to have a methemoglobin level of 24.2% on arterial blood gas and CT chest negative for pulmonary embolus. Methylene blue (100 mg intravenous) was administered with near immediate improvement in oxygenation and headache.

Upon hospital admission to the internal medicine service, the patient was started on nitrofurantoin for a urinary tract infection and did not require additional doses of methylene blue. Her methemoglobin level improved to 5.6% the following day. Her oxygenation remained appropriate throughout the hospitalization. The patient was discharged with resolution of symptoms and has remained symptom free.

## Discussion

Phenazopyridine-induced methemoglobinemia is rarely seen in the emergency setting and few cases relating directly to this medication are cited in the literature [1–5]. Cases of drug-induced methemoglobinemia are more frequently related to use of local anesthetic agents and dapsone [5–10]. Medications that have been associated with acquired methemoglobinemia are listed in Table 1 [5–20]. Nevertheless, phenazopyridine is a commonly used over-the-counter medication for symptoms of urinary tract infection that can cause significant methemoglobinemia.

**Table 1:** Medications associated with acquired methemoglobinemia

Drug class	Medications implicated in cases of methemoglobinemia
Analgesics	Acetaminophen (generally in overdose or combination with other oxidative stresses), Buprenorphine-naloxone, Celecoxib, Ibuprofen, Phenazopyridine
Anesthetics	Benzocaine, Lidocaine, Prilocaine (including topical creams), Tetracaine
Antiemetics	Metoclopramide
Antimicrobials	Atovaquone, Dapsone, Sulfonamides (sulfanilamide, sulfamethoxazole, sulfapyridine, sulfathiazide), Quinolones (chloroquine, primaquine)
Vasodilators	Amyl nitrate, Nitric oxide, Nitroglycerine, Nitroprusside
Xenobiotics	Aniline dyes, Naphthalene, Nitrates (ex: well water contamination), Nitrites (ex: fermented meats), Silver nitrate, Trinitrotoluene

Methemoglobinemia can be congenital or acquired [10]. Congenital causes are seen in cytochrome b5 reductase deficiency or hemoglobin M disease. All patients with hereditary methemoglobinemia should avoid exposure to aniline derivatives, nitrates, and other agents that may precipitate methemoglobinemia.

Methemoglobin forms when the ferrous (Fe_2_+) irons of heme are oxidized to the ferric (Fe_3_+) state. In the ferric state, methemoglobin is unable to bind oxygen. In addition, the oxygen affinity of any remaining ferrous heme in the hemoglobin tetramer is increased. As a result, the oxygen dissociation curve is shifted, resulting in hypoxia, cellular asphyxiation, and resultant lactic acid production [9, 10].

Induced or acquired methemoglobin typically manifest as a drug reaction. Methemoglobinemia may be suspected by the presence of clinical cyanosis in the presence of a normal arterial pO_2_ on arterial blood gas. The blood in methemoglobinemia has been described as “chocolate-brown,” red, or brownish to blue in color, which, unlike deoxy-hemoglobin, does not change color despite the addition of oxygen. Clinical manifestations occur when methemoglobin production is accelerated beyond the capacity of intrinsic NADPH methemoglobin-reductase activity.

To reverse methemoglobinemia, ferric hemoglobin must be reduced to ferrous hemoglobin by either of two pathways. The primary pathway is the NADH-dependent reaction catalyzed by cytochrome b5 reductase [10]. The alternate pathway uses nicotinamide adenine dinucleotide phosphate (NADPH) generated by glucose-6-phosphate dehydrogenase (G6PD) in the hexose monophosphate shunt to reduce ferric hemoglobin to ferrous hemoglobin, allowing for return to normal oxygen delivery, but this is not significantly active in normal physiology [10]. Extrinsic electron acceptors, i.e., methylene blue or ascorbic acid, are required for the activation of this pathway [10, 20].

Symptoms of clinically significant methemoglobinemia are vague, including headache, fatigue, dyspnea, and lethargy. In asymptomatic patients, usually with methemoglobin levels less than 20%, no therapy other than discontinuation of the offending agent will be sufficient [10, 20]. Patients with acute acquired methemoglobinemia may be symptomatic at lower levels of methemoglobin. Methylene blue therapy is indicated in symptomatic patients, or if the methemoglobin level is greater than 20% of hemoglobin profile on arterial blood gas.

At methemoglobin levels greater than 40%, respiratory depression, altered consciousness, shock, seizures, and death may occur. Hyperbaric oxygen, blood transfusion, or exchange transfusion may be helpful for patients in extremis [20]. A further discussion of symptoms associated with increasing methemoglobin concentrations is shown in Table 2 [20].

**Table 2:** Symptoms observed at increasing levels of methemoglobin

Methemoglobin %	Symptoms
< 3% (Normal)	None
3–15%	Possibly asymptomatic, low saturations on pulse oximeter, gray skin
15–20%	“Chocolate-brown” blood, cyanosis
20–50%	Dizziness, syncope, headache, dyspnea, exercise intolerance, weakness
50–70%	Central nervous system depression, dysrhythmias, acidosis, seizures, coma
> 70%	Profound hypoxia, death

Methylene blue provides an artificial electron transporter for the ultimate reduction of methemoglobin via the NADPH-dependent pathway. The dose of 1 to 2 mg/kg is given intravenously over several minutes to avoid painful local irritation and the response is usually rapid, occurring over the next few minutes [10, 20]. Repeated dosing may be considered if the level of methemoglobin is still high or the patient remains symptomatic after 1 h, but retreatment is rarely necessary [10, 20]. If repeated treatment fails to significantly reverse methemoglobin levels, the oxidant stress may have not been fully isolated and decontamination of the skin must be assured and possible gut decontamination considered [20]. However, patients with sulfhemoglobinemia (a methemoglobinemia mimic) and those with NADPH methemoglobin reductase deficiency may not improve despite methylene blue administration. For refractory cases of methemoglobinemia, hyperbaric oxygen and exchange transfusions may be beneficial, but are cost and time intensive and should be considered in the most extreme of cases [20].

In patients with G6PD deficiency, methylene blue can induce a hemolytic crisis [20]. In areas with high prevalence of G6PD deficiency, ascorbic acid (vitamin C) can be given in lieu of methylene blue in order to reduce the chances of inducing a hemolytic crisis [20]. It should be noted that if methylene blue is available, that treatment with ascorbic acid is not indicated for patients without G6PD deficiency [20].

For patients requiring dapsone treatment in which methemoglobin is noted, cimetidine can help reduce the oxidative stress and should be administered along with methylene blue in cases of dapsone overdose [20].

## Conclusions

In the described case, the decision to treat was largely based on clinical grounds. While other causes for tachycardia and hypoxia were considered on her arrival to the facility, the likelihood of methemoglobinemia combined with the relatively low risk from presumptive treatment with methylene blue led the team to recommend treatment as both a diagnostic and therapeutic maneuver. Clinical suspicion was confirmed with laboratory testing once available and other life-threatening diagnoses such as pulmonary embolus subsequently ruled out in due course. Of the cases described in the literature, most are due to relatively young patients taking few other medications and with limited past medical histories. Though methemoglobinemia secondary to phenazopyridine is rare, this case shows that in patients with complex medical histories, it is important to remember that this common and easily accessible medication can precipitate life-threatening hypoxia while also assessing for other, more common potential causes. This case serves to emphasize the importance of a thorough history and physical—tools especially helpful when at a satellite facility with relatively limited resources compared to those available in a hospital-based ED.

## Abbreviations

ABG: Arterial blood gas
CT: Computed tomography
ED: Emergency department
G6PD: Glucose-6-phosphate dehydrogenase
NADH: Nicotinamide adenine dinucleotide
NADPH: Nicotinamide adenine dinucleotide phosphate

## References

[1] Banimahd F, Loo T, Amin M, Ahadiat OR, Chakravarthy B, Lotfipour SA (2016). Rare but important clinical presentation of induced methemoglobinemia. West J Emerg Med.

[2] Shahani L., Sattovia S. (2012). Acquired methaemoglobinaemia related to phenazopyridine ingestion. Case Reports.

[3] Shatila W, Clark A (2013). Unexplained hypoxia in a woman presenting with acute on chronic abdominal pain. Am J Med.

[4] Yu CH, Wang CH, Chang CC (2011). Chocolate-colored blood with normal artery oxygen: methemoglobinemia related to phenazopyridine. Am J Med Sci.

[5] Daly JS, Hultquist DE, Rucknagel DL (1983). Phenazopyridine induced methaemoglobinaemia associated with decreased activity of erythrocyte cytochrome b5 reductase. J Med Genet.

[6] Orr TM, Orr DL (2011). Methemoglobinemia secondary to over-the-counter Anbesol. Oral Surg Oral Med Oral Pathol Oral Radiol Endod.

[7] Ash-Bernal R, Wise R, Wright SM (2004). Acquired methemoglobinemia—a retrospective series of 138 cases at 2 teaching hospitals. Medicine.

[8] Kane GC, Hoehn SM, Behrenbeck TR (2007). Benzocaine-induced methemoglobinemia based on the Mayo Clinic experience from 28 478 transesophageal echocardiograms—incidence, outcomes, and predisposing factors. Arch Intern Med.

[9] Darling RC, Roughton FJW (1942). The effect of methemoglobin on the equilibrium between oxygen and hemoglobin. Am J Phys.

[10] Tintinalli JE, Stapczynski JS (2011). Tintinalli’s emergency medicine: a comprehensive study guide.

[11] Khemiri M, Labassi A, Bagais A (2010). Toxic methemoglobinemia due to ibuprofen: report of a pediatric case. J Emerg Med.

[12] Kobayashi T, Kawabata M, Tanaka S (2000). Methemoglobinemia induced by combined use of sodium nitrate and acetaminophen. Intern Med.

[13] Sharma N, Varma S (2003). Unusual life-threatening adverse drug effects with chloroquine in a young girl. J Postgrad Med.

[14] Mary AM, Bhupalam L (2000). Metoclopramide-induced methemoglobinemia in an adult. J Ky Med Assoc.

[15] Karadsheh NS, Shaker Q, Ratroat B (2001). Metoclopramide-induced methemoglobinemia in a patient with co-existing deficiency of glucose-6-phosphate dehydrogenase and NADH-cytochrome b5 reductase: failure of methylene blue treatment. Haematologica.

[16] Carmon-Fonseca J, Alvarez G, Maestre A (2009). Methemoglobinemia and adverse events in Plasmodium vivax malaria patients associated with high doses of primaquine treatment. Am J Trop Med Hyg.

[17] Nash SL, Oehme FW (1984). A review of acetaminophen's effect on methemoglobin, glutathione, and some related enzymes. Vet Hum Toxicol.

[18] Hahn IH, Hoffman RS, Nelson LS (2004). EMLA-induced methemoglobinemia and systemic topical anesthetic toxicity. J Emerg Med.

[19] Kaushik P, Zuckerman SJ, Campo NJ (2004). Celecoxib-induced methemoglobinemia. Ann Pharmacother.

[20] Price DP, Hoffman RS, Howland M, Lewin NA, Nelson LS, Goldfrank LR (2015). Methemoglobin inducers. Goldfrank's Toxicologic emergencies.

